# Epigenetics and transgenerational inheritance in domesticated farm animals

**DOI:** 10.1186/2049-1891-5-48

**Published:** 2014-10-23

**Authors:** Amanda Feeney, Eric Nilsson, Michael K Skinner

**Affiliations:** Center for Reproductive Biology, School of Biological Sciences, Washington State University, 99164-4236 Pullman, WA USA

**Keywords:** Environment, Epigenetics, Pig, Review, Transgenerational

## Abstract

Epigenetics provides a molecular mechanism of inheritance that is not solely dependent on DNA sequence and that can account for non-Mendelian inheritance patterns. Epigenetic changes underlie many normal developmental processes, and can lead to disease development as well. While epigenetic effects have been studied in well-characterized rodent models, less research has been done using agriculturally important domestic animal species. This review will present the results of current epigenetic research using farm animal models (cattle, pigs, sheep and chickens). Much of the work has focused on the epigenetic effects that environmental exposures to toxicants, nutrients and infectious agents has on either the exposed animals themselves or on their direct offspring. Only one porcine study examined epigenetic transgenerational effects; namely the effect diet micronutrients fed to male pigs has on liver DNA methylation and muscle mass in grand-offspring (F2 generation). Healthy viable offspring are very important in the farm and husbandry industry and epigenetic differences can be associated with production traits. Therefore further epigenetic research into domestic animal health and how exposure to toxicants or nutritional changes affects future generations is imperative.

## Introduction

Mendelian genetic theories have guided much of the biological research preformed in recent history. It has long been assumed that specific phenotypes arise only from DNA sequence. However, non-Mendelian inheritance patterns challenge these theories and suggest that an alternate process might exist to account for certain mechanisms of inheritance. Epigenetics provides a molecular mechanism that can account for these non-Mendelian observations [1–3]. Epigenetics research looks into modifications and inheritance patterns that do not involve changes in the DNA sequence, but do affect genome activity and gene expression [1–4]. There are four main mechanisms by which epigenetics can alter gene expression: DNA methylation, histone modification, chromatin structure, and non-coding RNA [1, 5]. Although the epigenetic processes are highly conserved among all species, the specific epigenomes are highly divergent between species. Modifications of these epigenetic processes can occur due to direct environmental exposure at critical periods in the development of the organism [1, 6–8]. Clearly any generation that has direct exposure to an environmental insult may be altered in some way. Recent research shows subsequent generations that were not present at the time of the exposure can still be affected due to epigenetic transgenerational inheritance, if exposure occurred during sensitive developmental windows for the germ cells [9]. Epigenetic transgenerational inheritance is defined as germline-mediated inheritance of epigenetic information between generations, in the absence of direct environmental influences, that leads to phenotypic variation [1, 9]. For example, if a pregnant animal is exposed to a toxicant during gonadal sex determination of the fetus then changes in fetal germ cell epigenetic programming may occur [8, 10]. Therefore these offspring and the gametes that will form the grand-offspring are directly exposed to the toxicant, and changes seen in these F1 and F2 generations are not transgenerational [11]. However, epigenetic changes in the F3 generation (great-grand-offspring) would be considered transgenerationally inherited. In contrast, if a male or non-pregnant female adult animal is subjected to an environmental exposure, then changes seen in the F2 generation or later are considered transgenerational [11]. Changes in DNA methylation in gametes that are transmitted to subsequent generations provide a mechanism for the inheritance of epigenetic information [12–14]. Non-coding RNA also appears to have a role in epigenetic transgenerational inheritance [15]. Much of the current research has used rodent models to demonstrate epigenetic changes after environmental insult, especially during pregnancy [8, 10]. Germline epigenetic transgenerational inheritance has also been shown in plants, flies, worms, and humans [10, 16–21].

Despite the amount of epigenetic and transgenerational epigenetic inheritance research being done on a multitude of mammal, insect, and plant models [8, 10, 16–21], a lack of research into these topics using farm animal models exists. This review will present the current epigenetic inheritance research and data using farm animal models (bovine, porcine, ovine, and gallus), Table 1. While much of the work has focused on the direct effects of environmental exposure to toxicants and nutrients, research into epigenetic transgenerational inheritance is limited. It is important that more epigenetic research be done in domesticated farm animals because of their close human relationships and potential for high pesticide exposure on farms. Pesticides have been shown to have dramatic transgenerational epigenetic effects on many animal models affecting the nervous system, reproductive and endocrine systems, and even causing cancer [9, 22]. Since hybrid vigour (i.e. heterosis) has been shown to be critical in breeding of domestic animals, and epigenetics has a critical role in hybrid vigour [23], epigenetic inheritance will be important in developing optimal domestic animal breeds. Considering overpopulation issues requiring a rise in food supply, there may be more efficient ways of detecting and promoting favorable selection using epigenetics to breed for a lower instance of animal disease.

**Table 1:** **Environmental epigenetics and epigenetic inheritance in domestic farm animals**

Environmental epigenetics and domestic farm animals		
Bovine	Context	Ref.
Mammary gland-specific hypomethylation of Hpa II sites flanking the bovine alpha S1-casein gene.	Epigenetic regulation of lactation.	[24]
DNA-remethylation around a STAT5-binding enhancer in the alphaS1-casein promoter is associated with abrupt shutdown of alphaS1-casein synthesis during acute mastitis.	Epigenetic regulation of lactation.	[25]
Transcriptome profiling of Streptococcus uberis-induced mastitis reveals fundamental differences between immune gene expression in the mammary gland and in a primary cell culture model.	Epigenetic regulation of lactation.	[26]
Conservation of methylation reprogramming in mammalian development: aberrant reprogramming in cloned embryos.	Epigenetic changes with assisted reproductive technologies	[36]
DNA methylation events associated with the suppression of milk protein gene expression during involution of the bovine mammary gland.	Epigenetic regulation of lactation.	[27]
Effect of maternal lactation during pregnancy	Epigenetic regulation of lactation.	[28]
Large offspring syndrome in cattle and sheep.	Epigenetic changes with assisted reproductive technologies	[33]
The production of unusually large offspring following embryo manipulation: Concepts and challenges.	Epigenetic changes with assisted reproductive technologies	[34]
Postnatal characteristics of calves produced by nuclear transfer cloning.	Epigenetic changes with assisted reproductive technologies	[35]
Epigenetic contribution to individual variation in response to lipopolysaccharide in bovine dermal fibroblasts.	Epigenetic role in immunity	[39]
Occurrence, absorption and metabolism of short chain fatty acids in the digestive tract of mammals.	Epigenetic changes with regulation of nutrition	[29]
Butyrate induces profound changes in gene expression related to multiple signal pathways in bovine kidney epithelial cells.	Epigenetic changes with regulation of nutrition	[30]
Transcriptome Characterization by RNA-seq Unravels the Mechanisms of Butyrate-Induced Epigenomic Regulation in Bovine Cells.	Epigenetic changes with regulation of nutrition	[31]
In vitro produced and cloned embryos: Effects on pregnancy, parturition and offspring.	Epigenetic changes with assisted reproductive technologies	[32]
**Porcine**		
Sulforaphane causes a major epigenetic repression of myostatin in porcine satellite cells.	Histone deacetylase inhibitor affects myostatin *in vitro*	[41]
Maternal dietary protein affects transcriptional regulation of myostatin gene distinctively at weaning and finishing stages in skeletal muscle of Meishan pigs.	Maternal diet affects offspring epigenetics	[43]
HOX10 mRNA expression and promoter DNA methylation in female pig offspring after in utero estradiol-17beta exposure.	Maternal steroid exposure affects offspring epigenetics	[48]
Investigations on transgenerational epigenetic response down the male line in F2 pigs.	Paternal diet has transgenerational epigenetic effect	[49]
Dietary Sulforaphane, a Histone Deacetylase Inhibitor for Cancer Prevention	Epigenetic changes with regulation of nutrition	[40]
Neonatal estradiol exposure alters uterine morphology and endometrial transcriptional activity in prepubertal gilts.	Steroid exposure affects epigenetics	[47]
Maternal dietary protein restriction and excess affects offspring gene expression and methylation of non-SMC subunits of condensin I in liver and skeletal muscle.	Maternal diet affects offspring epigenetics	[44]
Diet, methyl donors and DNA methylation: interactions between dietary folate, methionine and choline.	Epigenetic changes with regulation of nutrition	[45]
**Ovine**		
Periconceptional nutrition and the early programming of a life of obesity or adversity.	Maternal diet affects offspring epigenetics	[50]
The effect of maternal under-nutrition before muscle differentiation on the muscle fiber development of the newborn lamb.	Maternal diet affects offspring epigenetics	[51]
Effect of maternal dietary restriction during pregnancy on lamb carcass characteristics and muscle fiber composition.	Maternal diet affects offspring epigenetics	[52]
**Gallus**		
Comparison of the Genome-Wide DNA Methylation Profiles between Fast-Growing and Slow-Growing Broilers.	Differences in epigenetics between breeds	[55]
Insulin-like growth factor-1 receptor is regulated by microRNA-133 during skeletal myogenesis.	Epigenetic effects during muscle development	[58]
Transgenerational epigenetic effects on innate immunity in broilers: An underestimated field to be explored?	Review on role of epigenetics in innate immunity	[59]
DNMT gene expression and methylome in Marek's disease resistant and susceptible chickens prior to and following infection by MDV.	Epigenetic role in immunity	[54]
**Epigenetic transgenerational inheritance study in domestic farm animals**		
**Porcine**		
Investigations on transgenerational epigenetic response down the male line in F2 pigs.	Paternal diet has transgenerational epigenetic effect	[49]

## Domestic animal models

### Bovine

The relationship of DNA methylation and milk production in dairy cattle has been investigated. During lactation the bovine αS1-casein gene is hypomethylated [24]. Research has characterized this gene during various physiological conditions during the lactation cycle. Vanselow *et al*. found that during lactation the (STAT)5-binding lactation enhancer, which is part of the αS1-casein encoding gene, is hypomethylated [25]. However, during *Escherichia coli* infection of the mammary gland, this region becomes methylated at three CpG dinucleotides which accompanies a shut down of αS1-casein synthesis [25]. These observations have also been shown with infection by *Streptococcus uberis*[26]. In addition, methylation of these same 3 CpG dinucleotides has been seen during non-milking periods of healthy dairy cattle when milking was ceased suddenly [27]. González-Recio *et al*. preformed a generational study to see if a mother dairy cow affected the milk production of her offspring [28]. They found that female calves born to cows that were already lactating from previous births produced between 18 and 91 kg less milk in adulthood than calves that were first-born, and that their lifespans were also shorter [28]. Because of the generational effect, researchers suggested epigenetic inheritance. However, they did not look specifically at epigenetic differences in the affected calves versus controls.

More research has been done on histone modification related to nutritional changes than on DNA methylation. Short-chain fatty acids are particularly important in ruminant digestion, and are used for cell energy production and use [29]. Butyrate, a specific short-chain fatty acid, inhibits histone deacetylases which have been shown to regulate epigenetic changes to the genome [30]. Wu, *et al*. [31] show that high doses of butyrate exposure to Madin-Darby bovine kidney epithelial cells causes cell cycle arrest, changes in gene expression, changes in nucleic acid metabolic processes, regulation of the cell cycle, and changes in DNA replication. Therefore this study claims that histone acetylation is essential for diverse cellular processes [31], but histone acetylation was not measured directly.

The influence of epigenetics on disease has been studied in many animal models such as rats, mice, and humans, but very little has been done with cattle. One bovine developmental disease called large-offspring syndrome (LOS) has been found to have epigenetic components during embryonic growth. LOS has largely been associated with reproductive technologies commonly used with cattle such as in vitro fertilization and somatic-cell nuclear transfer [32]. Symptoms usually include increases in birth weight, organ overgrowth, difficulty breathing and standing, as well as skeletal and immunological defects. There are also increased rates of fetal and neonatal deaths [33–35]. Dean *et al*. [36] has reported methylation changes in bovine embryos (morulae) between controls, *in vitro* fertilized, and somatic-cell nuclear transfer embryos, and suggests that these methylation differences may account for the different success rates and health of calves born from these reproductive technologies [36]. A number of studies have demonstrated developmental epigenetic programming in bovine germ cells [37] and bovine embryos [38], which is similar among all mammalian species. In another study focusing on innate immunity, Green *et al*. [39] looked at epigenetics and individual variation in the innate immune response of bovine dermal fibroblasts, specifically via toll receptor signaling. Exposure to de-methylating and hyper-acetylating agents led to increased expression of several cytokines as compared to controls, suggesting immune gene expression has epigenetic regulation [39].

No studies have been published showing epigenetic transgenerational inheritance in cattle.

### Porcine

Swine are often used as animal models to study human disease because of the similar physiology between the two species. Because of this, much of the epigenetic porcine research involves exposure and response, with very little of the current research being transgenerational.

Epigenetic effects due to histone modification and acetylation have been studied in a porcine model both in order to increase meat production and to develop a potential treatment for muscular degenerative disease. Sulforaphane is a bioactive histone deacetylase inhibitor often found in edible vegetation like broccoli [40]. Fan *et al*. [41] treated porcine satellite cells with sulforaphane to epigenetically repress myostatin which would potentially result in more muscle growth [42]. Liu *et al*. [43] also looked at the myostatin pathway to investigate the short term and long term epigenetic changes in pigs based on maternal diet. These researchers concluded that histone modifications and changes in microRNA expression took place long term and played a part in skeletal muscle phenotype [43]. Another study looked at DNA methylation in response to altered protein and carbohydrate diets for maternal pigs during gestation [44]. Researchers found that hepatic global methylation was decreased in fetuses from protein-restricted mothers, likely caused by methionine deficiency [45]. However, skeletal muscle global methylation was not affected [44]. This study demonstrates maternal nutrition will likely have an epigenetic effect on embryonic tissue development. Epigenetic programming in the porcine germline has also been reported [46].

Research conducted by Tarletan *et al*. demonstrated that neonatal estrogen exposure in piglets can lead to epigenetic changes that affect uterine capacity and environment [47]. This leads to potentially less successful pregnancies once the piglets become adults [47]. Another environmental estrogen exposure experiment was preformed analyzing the effect on the gene HOXA10 by exposing offspring in utero to estradiol-17β. No difference in HOXA10 expression was detected in either the low dose or high dose group [48]. However, differences in HOXA10 mRNA expression were detected between pre-pubescent and post-pubescent gilts [48].

One recent transgenerational porcine study has been reported [49], Table 1. Braunschweig *et al*. preformed a three generational study to look at the effect of feeding on male epigenetic inheritance. The experimental group F0 generation males were fed a diet high in methylating micronutrients, and the resulting F2 generation had a lower fat percentage and higher shoulder muscle percentage as compared to controls. They also found significant differences in DNA methylation between the control and experimental groups, especially in the liver, which was proposed to epigenetically affect fat metabolism pathways [49].

### Ovine

As shown in the bovine model and porcine model, maternal nutritional impact is a common topic in epigenetic research, and ovine studies are no exception. Zhang *et al*. [50] looked into the effects of maternal over-nutrition in sheep, both during peri-conception and during the late stages of pregnancy. They found that over-nutrition in late stages of pregnancy resulted in more visceral fat in offspring and a change in appetite that pre-disposed that lamb to over-eat in adult life. More interestingly, they also found that over-nutrition at the peri-conception period led to higher rates of visceral fat in only female ewe offspring, leading to a conclusion of sex-specific DNA methylation. They also found that when diet was restricted just before conception (maternal under-nutrition), the adrenal glands of the offspring tended to be heavier and have less methylation of the IGF2/H19 differentially methylated regions in the adrenal. Observations suggested that while a restricted peri-conception diet led to no maternal epigenetic influence on bodyweight, it did increase the stress response in these offspring [50]. Other nutritional studies have looked at muscle development in response to maternal under-nutrition during pregnancy and have shown that maternal under-nutrition causes a decrease of fast muscle fibers in early stages, but an increase in them during later stages of development [51, 52]. However, these studies did not investigate epigenetic mechanisms.

No studies have been published showing epigenetic transgenerational inheritance in sheep.

### Gallus

Marek’s disease in chickens is a manifestation of Marek’s disease virus and progresses to become a T-cell lymphoma that affects chickens and other birds. Vaccines have been developed but they are not completely successful [53]. Tian *et al*. [54] set to find out why one breeding line seemed resistant to the virus, while another was more susceptible. They found that in the virus-resistant line, DNA methylation levels in thymus cells were decreased after exposure to the virus. They also found that with pharmacological inhibition of DNA methylation *in vitro* the propagation in the infected cells was slowed. Observations suggested that DNA methylation in the host may be associated with virus resistance or susceptibility [54].

Different developmental epigenetic patterns have been studied between chicken types. One study looked at differential DNA methylation in breast muscle between slow-growing and fast-growing broiler chickens [55]. They found that between the two breeds of chickens there were 75 differentially methylated genes, including several genes belonging to the fibroblast growth factor (FGF) family. The FGF family is known for its role in many growth processes [56]. In addition, effects in the insulin growth factor receptor (IGF1R) were observed that influence skeletal muscle growth specifically [57, 58].

As one review indicated, many poultry studies indicate that there may be epigenetic effects, and even transgenerational epigenetic inheritance, though very few studies actually test for DNA methylation or histone modification in their research [59].

No studies have been published showing epigenetic transgenerational inheritance in chicken.

## Conclusion

While a good amount of epigenetic research has been preformed on domesticated farm animals still more needs to be done, Table 1. There is little research at all in transgenerational inheritance of these epigenetic modifications. This could be due to the fact that farm animals are more difficult and more costly to raise than other common animal research models. In addition, they have longer lifespans so transgenerational studies take more time and resources. Animal science researchers should cultivate an interest in conducting these types of experiments for a number of reasons. Healthy viable offspring are very important in the farm and husbandry industry and epigenetic differences can be associated with production traits. Recently there has been a lot of social pressure to cut down on vaccination and antibiotic use for animals raised for meat and epigenetics research may help to provide the key to lowering disease and increasing immunity. Therefore research into domestic animal health and how exposure to toxicants such as pesticides affects future generations is imperative.

### Glossary

Epigenetics: Molecular factors/processes around the DNA that regulate genome activity independent of DNA sequence, and are mitotically stable.

Epigenetic: Transgenerational Inheritance: Germline-mediated inheritance of epigenetic information between generations in the *absence* of direct environmental influences, that leads to phenotypic variation.

Epimutation: Differential presence of epigenetic marks that lead to altered genome activity.

## Abbreviations

F0: Generation pregnant female
F1: Generation fetus that becomes the offspring or children
F2: Generation (grandchildren)
F3: Generation (great-grandchildren)
LOS: Large-offspring syndrome
FGF: Fibroblast growth factor
IGF1R: Insulin growth factor receptor.
